# Adenoid cystic carcinoma of the parotid metastasizing to liver: case report

**DOI:** 10.1186/1471-2407-4-41

**Published:** 2004-07-30

**Authors:** K Harish, SR Mangala Gouri

**Affiliations:** 1 Department of Surgical Oncology, M. S. Ramaiah Medical College & Hospital, Bangalore – 560054, India; 2 Department of Pathology, M. S. Ramaiah Medical College & Hospital, Bangalore – 560054, India

## Abstract

**Background:**

Adenoid cystic carcinoma is a rare malignant parotid tumor. Metastasis can occur even a decade or more after initial treatment of the primary.

**Case presentation:**

We report a 60 year old female patient who presented with adenoid cystic carcinoma of the parotid gland. She underwent a total conservative parotidectomy followed by adjuvant radiotherapy. While on follow up, patient developed multiple liver metastases which manifested three years later. Patient lived for another two years before she died of her disease.

**Conclusions:**

Although distant metastases of adenoid cystic carcinoma develop frequently, isolated metastasis to liver is unusual. Even after manifestation of distant metastasis, patients can be expected to live for a number of years. Palliative chemotherapy can be considered in symptomatic cases while the usefulness of metastatectomy is controversial.

## Background

Adenoid cystic carcinoma (ACC) is a rare malignant neoplasm of the salivary gland. Salivary gland neoplasms constitute 3% of cancers of all sites, of which, 10–15% are malignant [1,2]. Though ACC is the most common malignant tumor of the submandibular, sublingual and minor salivary glands, it accounts for only 15% of parotid cancers [3]. They are generally slow growing and spread relentlessly to adjacent structures. Hematogenous spread is more common than lymphatic spread, the common sites of metastasis being the lung, bone and viscera [4,5]. We present a case of multiple liver metastases occurring 3 years after surgery for ACC of the parotid gland. The primary therapy, metastasis and outcome of ACC are discussed.

## Case presentation

**Figure 1:**
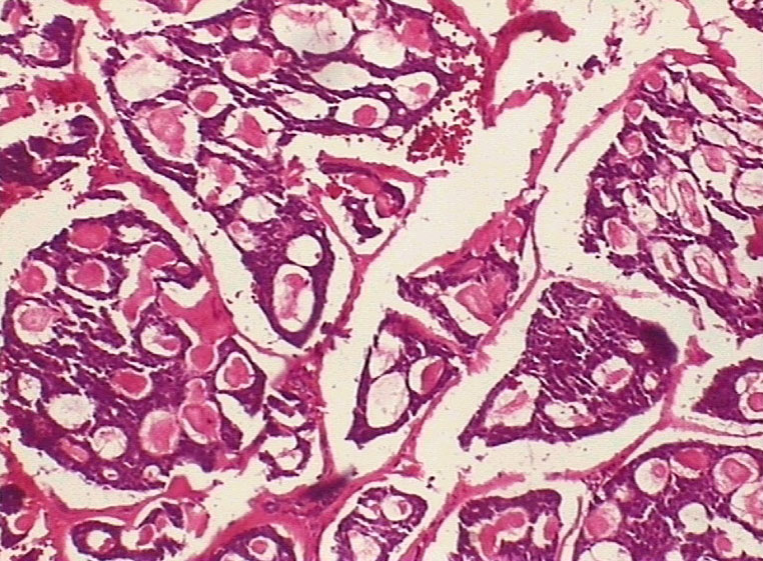
Photomicrograph showing small darkly stained cells with scanty cytoplasm arranged in nests fenestrated by round or oval spaces – cribriform pattern, H & E (× 100)

**Figure 2:**
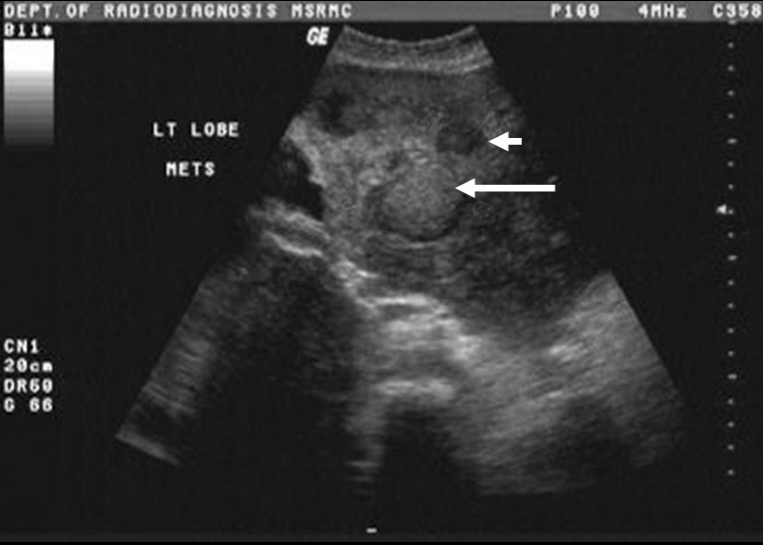
Abdominal ultrasound showing multiple hypoechoiec (short arrow) and hyperechoeic lesions (long arrow) in the liver

**Figure 3:**
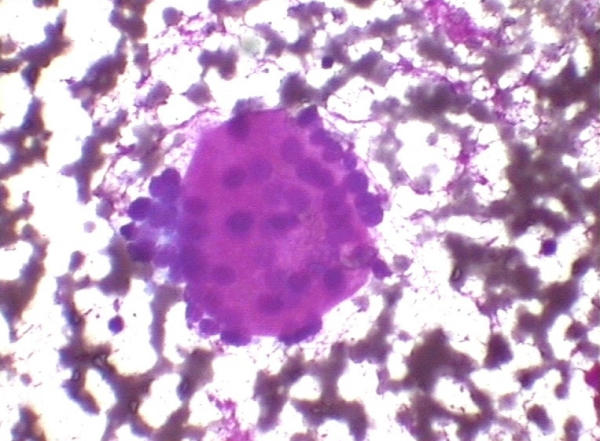
FNAC liver showing metastatic ACC, H & E (× 400)

A 60 year old woman presented with a small swelling beneath the right ear lobe of 4 months duration. The swelling measured 2 × 1 cm placed in the superficial part of the parotid and was not fixed. There was no facial nerve palsy or palpable cervical nodes. A fine needle aspiration cytology (FNAC) was carried out which showed the lesion to be ACC [6]. The clinical staging was T1, N0, M0. The patient underwent a total conservative parotidectomy after metastatic work up. Histopathology revealed ACC with cribriform pattern and perineural invasion (Figure 1). 60 Gy adjuvant external beam radiotherapy was administered post-operatively to the parotid area and the neck. Patient was placed on regular follow-up. Three years after primary surgery, patient presented with heaviness and pain in the right hypochondrium of 15 days duration. Patient was anicteric and abdominal examination revealed firm nodular and non tender enlargement of the liver. There was no ascites. Surgical site and neck were clinically normal. Chest roentgenogram was normal. Ultrasonography (US) of the abdomen revealed multiple metastatic lesions scattered in both lobes (Figure 2). Liver function tests were normal and an US guided FNAC revealed metastatic ACC (Figure 3). Since the lesions were multiple and scattered over both lobes of liver, surgical option was not considered and the patient was offered palliative chemotherapy which she declined. She developed pedal edema and abdominal distention 20 months after detection of liver metastasis. On clinical examination, patient was anicteric but liver had increased in size and abdomen showed evidence of a little free fluid. Chest CT scan was normal. Bone scan did not suggest any metastatic focus. Patient died a month later still without evidence of local recurrence or pulmonary metastasis.

## Conclusions

Although ACC is the second most common malignant salivary gland neoplasm and constitutes approximately one third of all salivary gland malignancies it constitutes only 2% of parotid neoplasms [3]. As ACC is neurotropic, frozen section analysis of nerve margins is suggested specially when nerve is grossly involved by the tumor [7]. A total conservative or a radical parotidectomy is advocated for ACC though the main intent is to obtain a tumor free area of at least 1 cm [8]. ACC, with its often unusually slow biologic growth, tends to have a protracted course and ultimately a poor outcome, with a 10-year survival reported to be less than 50% for all grades [9,10]. These carcinomas typically show frequent recurrences and late distant metastases [11]. In a retrospective review of 92 cases, a tumor size greater than 4 cm was associated with an unfavorable clinical course [12]. Cribriform and solid patterns seen histologically were thought to predict more biological aggressiveness while tubular pattern represented more differentiated pattern of ACC. Over long periods of patient follow-up such grade based prognostication is less valid. Currently, stage and tumor location are the only factors considered prognostically significant [13].

Radiotherapy has been used as a primary modality for patients with surgical contraindications and in those with unresectable neoplasms. Though no improvement in survival is reported, the use of adjuvant radiation improves locoregional control and disease free survival. This patient received adjuvant radiation and did not have any locoregional recurrence.

Regional metastasis is less common occurring in about 17% while systemic failure occurs in 33 to 50% of the patients [4,8]. Though involvement with distant metastases are unpredictable, organs involved in the order of decreasing frequency are lung, bone, brain and the liver [3]. Other rare metastatic sites of parotid and non parotid ACC include stomach, toe, choroids, brain and skin [14-18]. The initial site of metastasis is usually the organ containing the first capillary bed (first filter) and hence lungs would be the common site of metastasis [19]. Clinical observations from various malignancies have indicated that metastasis from certain types of tumor tend to occur in specific target organs leading to the famous 'soil and seed' hypothesis where metastatic cells 'home' to the organ [20]. Though liver metastasis has been reported, most of the liver metastases reported are of non parotid ACC [3,5]. The occurrence is usually metachronous or synchronous with metastasis to other organs like the lung as it is the first filter. In the series of Spiro, of the 74 patients developing metastasis from salivary gland ACC, 23 did so without loco-regional recurrence while 5 had isolated bone metastasis [5]. Sung and colleagues found metastases in 46 out of 94 head and neck ACC [21]. In that study, only one patient developed liver metastasis and that patient had metastasis to both lung and bone. In this case, the patient manifested with multiple metastatic foci in the liver as the first and only metastatic organ which is very unusual. Surgical options of metastatectomy were not explored as patient had multiple metastases involving both lobes of liver. Although the patient lived with disease for a further two years, she did not show any evidence of lung metastasis or loco-regional recurrence. In this patient, the liver metastasis could have occurred prior to treatment as an organ of preference; evidenced by the fact that there were no other organs showing metastasis nor was there a loco-regional recurrence later. Studies in ACC have shown long tumor doubling times of pulmonary metastasis and late recurrences up to 10 years after primary treatment [22]. The estimated doubling time of lung metastasis in ACC ranges from 200 to 600 days [22]. There is even a suggestion that metastasis at the cellular level could occur many years prior to clinical presentation of primary tumor. FNAC was done to prove the metastatic foci in this patient and can be a useful tool for diagnosis [23]. Although this patient declined chemotherapy, chemotherapeutic responses have been reported in ACC [24]. ACC carries a mortality of 75–80% over a 30 year period and most patients who die of their disease do so between 5 and 10 years after initial treatment [10]. Our patient died 5 years from diagnosis with metastatic disease that developed 3 years after initial treatment.

ACC is a rare malignant tumor of parotid gland. Metastasis can manifest very late and hence a long term follow-up and a high index of suspicion is necessary to diagnose them early. An annual ultrasound study of abdomen would be desirable on follow-up. Unlike metastasis from other malignancies, these grow indolently and long term survival can be expected even with multiple metastases as also evidenced in the present case. Chemotherapy could be considered in selected patients as a therapeutic option in metastatic disease.

## List of abbreviations used

**ACC: **Adenoid cystic carcinoma

**FNAC: **Fine needle aspiration cytology

**Gy: **Gray

## Competing interests

None declared

## Authors' contributions

**KH **was the principal clinician who planned the evaluation and procedure, in addition to conceptualizing and drafting the article.

**SRMG **was the pathologist.

Both the authors read and approved the final manuscript.

## Pre-publication history

The pre-publication history for this paper can be accessed here:
